# Global burden and trend of tuberculosis in children and adolescents (under 15 years old) from 1990 to 2021, with projections to 2040

**DOI:** 10.3389/fpubh.2025.1578658

**Published:** 2025-06-25

**Authors:** Yuanhao Liang, Jiayi Wang, Jianzhou Yang, Jinjia Liu, Xiaofeng He

**Affiliations:** ^1^Clinical Experimental Center, Jiangmen Key Laboratory of Clinical Biobanks and Translational Research, Jiangmen Central Hospital, Jiangmen, China; ^2^Queen Mary College, Nanchang University, Nanchang, China; ^3^Department of Public Health and Preventive Medicine, Changzhi Medical College, Changzhi, China; ^4^Department of Biochemistry, Changzhi Medical College, Changzhi, China; ^5^Institute of Evidence-Based Medicine, Heping Hospital Affiliated to Changzhi Medical College, Changzhi, China

**Keywords:** global burden of disease, tuberculosis, children and adolescents, age-standardized rate, estimated annual percentage change

## Abstract

**Background:**

Tuberculosis (TB) remains a significant global health issue, but its burden among children and adolescents under 15 years old is not well quantified. This study evaluates TB trends in this age group from 1990 to 2021 and projects future trends through 2040.

**Methods:**

We used data from the Global Burden of Disease Study (GBD) 2021 to assess the incidence and mortality of TB in children and adolescents (under 15) from 1990 to 2021. A Bayesian age-period-cohort model was employed to project the TB burden.

**Results:**

In 2021, there were 799,047 new TB cases and 81,870 TB-related deaths among children, with an age-standardized incidence rate (ASIR) of 40.01 per 100,000 population and an age-standardized mortality rate (ASMR) of 4.16 per 100,000 population. From 1990 to 2021, the ASIR declined by 2.4% annually, while ASMR decreased by 4.19% per year. However, drug-resistant TB, especially extensively drug-resistant TB, increased significantly. The burden was highest in low-SDI regions, particularly among children under 5, who accounted for over 75% of TB-related deaths. Projections to 2040 indicate continued declines in ASIR and ASMR for all TB forms, including drug-resistant and TB-HIV co-infections.

**Conclusion:**

Sustained investment in TB control programs, particularly in low-SDI regions, is crucial. Addressing drug-resistant TB and TB-HIV co-infection should be prioritized in global public health strategies.

## 1 Introduction

Tuberculosis (TB) remains a major global public health concern, ranking among the leading infectious causes of morbidity and mortality worldwide (1). While TB predominantly affects adults, children and adolescents also represent a substantial and often under-recognized portion of the global TB burden (2). Each year, an estimated 1 million children under the age of 15 develop TB, with many cases going undetected due to non-specific symptoms and a lack of age-appropriate diagnostic tools (3). Adolescents, who undergo physiological and immunological changes during puberty, are at an increased risk of developing active TB, which frequently goes unnoticed and untreated (2).

The epidemiology of TB in children and adolescents varies widely across regions, with socioeconomic factors, HIV co-infection, malnutrition, and access to healthcare services playing pivotal roles (4, 5). In low- and middle-income countries, particularly where TB transmission rates are high, children and adolescents are often exposed to the disease at a young age (6). However, the true burden of TB in these populations is likely underreported due to diagnostic limitations, under-resourced healthcare systems, and the lack of targeted TB interventions for younger age groups (7). As a result, children and adolescents are particularly vulnerable to undiagnosed and untreated TB, which can lead to severe disease progression and further transmission within communities (8). As a result, enhancing TB prevention, detection, and treatment in pediatric populations has become a key focus in global TB control efforts.

The World Health Organization (WHO) has set a goal to eliminate TB by 2035 through its End TB Strategy (9). Achieving this target necessitates addressing TB across all age groups, with a special focus on susceptible populations such as children and adolescents (5). By identifying trends in the TB burden among younger populations, policymakers and public health experts can prioritize interventions aimed at these high-risk groups. TB in children and adolescents is often complicated by co-morbidities like malnutrition, HIV, and other infectious diseases, further hindering diagnosis and treatment (10). A global assessment of the TB burden can help pinpoint regions where these co-morbidities are prevalent, allowing for the development of integrated healthcare approaches (11). Therefore, assessing the global TB burden and trends among children and adolescents is crucial.

This study aims to provide a thorough analysis of the global, regional, and national trends of TB among children and adolescents under the age of 15, emphasizing the scale of the burden and the critical need for targeted public health interventions. By exploring the epidemiological patterns of TB in these age groups, we seek to contribute to the development of more effective strategies for TB prevention, diagnosis, and treatment.

## 2 Methods

### 2.1 Overview

The Global Burden of Diseases Study (GBD) is a large-scale collaborative research effort aimed at estimating morbidity and mortality across a wide range of diseases, injuries, and risk factors. The GBD Collaborator Network comprises over 10,000 contributors worldwide (1). With each new edition of the GBD, data are updated, and new methods are introduced. As a result, estimates for the entire time series replace those previously reported in earlier GBD rounds. In this study, we estimated the epidemiological characteristics and temporal trends of tuberculosis among children and adolescents under 15 years of age, analyzing by age, socio-demographic index, and geographic location from 1990 to 2021. The GBD protocol received approval from the University of Washington’s research ethics board and will be conducted in full compliance with university policies, as well as applicable federal, state, and local regulations (1). This study was carried out following the GBD protocol, and informed consent was waived due to the use of deidentified data.

### 2.2 Case definition

The case definition includes all forms of TB, including pulmonary TB and extrapulmonary TB, which are bacteriologically confirmed or clinically diagnosed. For TB, the ICD-10 codes are A10-A19.9, B90-B90.9, K67.3, K93.0, M49.0, P37.0, and ICD-9 codes are 010–019.9, 137–137.9, 138.0, 138.9, 139.9, 320.4, 730.4–730.6. For HIV-TB, the ICD-10 code is B20.0. In the GBD 2021, TB incidence was estimated separately according to drug sensitivity results and HIV status. The case definitions are shown below.

1. Drug-susceptible TB (DS-TB): TB (among HIV-negative individuals) that is susceptible to isoniazid and rifampicin.
2. Multidrug-resistant TB without extensive drug resistance (MDR-TB): a form of TB (among HIV-negative individuals) that is resistant to the two most effective first-line anti-tuberculosis drugs (isoniazid and rifampicin) but is not resistant to any fluoroquinolone and any second-line injectable drugs (amikacin, kanamycin, or capreomycin).
3. Extensively drug-resistant TB (XDR-TB): a form of TB (among HIV-negative individuals) that is resistant to isoniazid and rifampicin, plus any fluoroquinolone and any second-line injectable drugs.
4. HIV/AIDS – drug-susceptible TB (HIV-DS-TB): TB (among HIV-positive individuals) that is susceptible to isoniazid and rifampicin.
5. HIV/AIDS – multidrug-resistant TB without extensive drug resistance (HIV-MDR-TB): a form of TB (among HIV- positive individuals) that is resistant to the two most effective first-line anti-tuberculosis drugs (isoniazid and rifampicin) but is not resistant to any fluoroquinolone and any second-line injectable drugs (amikacin, kanamycin, or capreomycin).
6. HIV/AIDS – extensively drug-resistant TB (HIV-XDR-TB): a form of TB (among HIV-positive individuals) that is resistant to isoniazid and rifampicin, plus any fluoroquinolone and any second-line injectable drugs.

### 2.3 Data collection

Data on the annual incidence and deaths from TB between 1990 and 2021 for children and adolescents under 15 years of age across 204 countries and territories were collected using the Global Health Data Exchange (GHDx) query tool.^1^ The GBD 2021 grouped these 204 countries and territories into 21 regions based on epidemiological similarities and geographic proximity. Additionally, the Socio-demographic Index (SDI) was also used to categorize regions and countries into five quintiles: low-SDI (0–0.455), low-middle-SDI (0.455–0.608), middle-SDI (0.608–0.690), high-middle-SDI (0.690–0.805), and high-SDI (0.805–1). The SDI is a composite measure that reflects social and economic conditions influencing health outcomes, calculated using national income per capita, average years of education for individuals over 15, and the total fertility rate among women under 25. In this study, the SDI was employed to examine the relationship between regional development level and the incidence or mortality of TB.

### 2.4 Statistical analysis

The age-standardized rates (ASR) of incidence and mortality for TB per 100,000 population were calculated by employing the following formula:


$$\mathrm{ASR}=\frac{\sum_{i=1}^{A}a_{i}w_{i}}{\sum_{i=1}^{A}w_{i}}\times 100,000$$

Where $a_{i}$ denotes the $i^{\mathrm{th}}$ age subgroup and the number of persons (or weight) ( $w_{i}$ ) in the same age class *i* of the chosen reference standard population. The value was then divided by the sum of standard population weights. The population counts for each age group were obtained from the GBD Study Population Estimates for the years 1950–2021 (12). It is assumed that the natural logarithm of ASR changes is linear over time, represented by the equation y=α+βx+ε , where y=ln(ASR) and x is the calendar year and ε is the error term. The estimated annual percentage changes (EAPCs) in ASRs were calculated using the formula 100×(exp(β)−1) , with the corresponding 95% confidence interval (CI) derived from the linear regression model (13). The ASR was considered to be increasing if both the Estimated Annual Percentage Change (EAPC) and the lower bound of its 95% confidence interval (CI) were positive. Conversely, the ASR was regarded as decreasing if both the EAPC estimate and the upper bound of its CI were negative.

Moreover, Pearson correlation analysis was performed to assess the relationship between ASR and the SDI, with the results visualized using Locally Weighted Scatterplot Smoothing (LOWESS) curves. Furthermore, the ASR of incidence and mortality for TB among children and adolescents under 15 years of age from 2022 to 2040, was projected using the Bayesian age-period-cohort (BAPC) model with nested Laplace approximations (14). The BAPC model calculates hypothetical probability distributions based on three factors—age, period, and cohort—and integrates both prior and sample information to derive posterior distributions. This model is more flexible in selecting parameters and prior probability distributions, resulting in more robust and reliable predictions (15). Notably, projections using the BAPC model are based on the continuation of historical trends observed between 1990 and 2021. Implicit in these projections is the assumption that key influencing factors—such as TB program funding, healthcare infrastructure, diagnostic and treatment accessibility, population health behaviors, and demographic transitions—will remain relatively stable or improve gradually over time. The global age-standardized population data were sourced from the World Standards database developed by the WHO,^2^ and population forecast data were obtained from the GBD Global Fertility, Mortality, Migration, and Population Forecasts for 2017–2,100 (16). The “BAPC” R package facilitates the application of the BAPC model, enabling the generation of well-calibrated probabilistic forecasts with relatively narrow uncertainty intervals. All statistical analyses and mapping were conducted using R software, version 4.1.0 (R Foundation for Statistical Computing), with statistical significance defined as *p* < 0.05.

## 3 Results

### 3.1 Global and regional levels

In 2021, there were 799,047 new cases of TB among children and adolescents under 15 years old, with an age-standardized incidence rates (ASIR) of 40.01 (95% uncertainty interval [UI] 39.92 to 40.1) per 100,000 population (Table 1). Globally, TB caused 81,870 deaths in this group, there were 81,870 deaths from TB, and the age-standardized mortality rates (ASMR) was 4.16 (95% UI 4.13 to 4.19) per 100,000 population. From 1990 to 2017, the global ASIR decreased annually on average by 2.4% (95% CI 2.18 to 2.62%), while the global ASMR decreased by 4.19% (95% CI 3.71 to 4.66%) annually. However, both the ASIR and ASMR for drug-resistant TB, particularly for extensively drug-resistant cases, regardless of HIV status, increased significantly in this population worldwide between 1990 and 2021 (Table 1).

**Table 1:** Incidence and deaths of tuberculosis among children and adolescents under the age of 15 in 1990 and 2021, and their estimated annual percentage changes from 1990 to 2021.

Characteristics	Incidence					Deaths				
	Number of cases in 1990	Age-standardized rate per 100,000 population, 1990	Number of cases in 2021	Age-standardized rate per 100,000 population, 2021	Estimated annual percentage change, 1990–2021	Number of cases in 1990	Age-standardized rate per 100,000 population, 1990	Number of cases in 2021	Age-standardized rate per 100,000 population, 2021	Estimated annual percentage change, 1990–2021
Global	1,459,600	82.51 (82.38 to 82.64)	799,047	40.01 (39.92 to 40.1)	−2.4 (−2.62 to −2.18)	294,850	16.05 (15.99 to 16.11)	81,870	4.16 (4.13 to 4.19)	−4.19 (−4.66 to −3.71)
Causes										
DS-TB	1,420,310	80.37 (80.24 to 80.5)	725,593	36.35 (36.27 to 36.44)	−2.61 (−2.71 to −2.51)	273,170	14.88 (14.82 to 14.94)	64,518	3.28 (3.26 to 3.31)	−4.56 (−4.75 to −4.36)
MDR-TB	8,086	0.46 (0.45 to 0.47)	32,515	1.63 (1.61 to 1.65)	1.28 (0.01 to 2.57)	2,120	0.12 (0.11 to 0.12)	5,887	0.3 (0.29 to 0.31)	0.64 (−0.87 to 2.19)
XDR-TB	0	0 (0 to 0)	1,193	0.06 (0.06 to 0.06)	10.04 (7.35 to 12.8)	0	0 (0 to 0)	253	0.01 (0.01 to 0.01)	11.55 (6.95 to 16.35)
HIV-DS-TB	31,071	1.68 (1.66 to 1.7)	37,628	1.86 (1.85 to 1.88)	−0.42 (−2.22 to 1.42)	19,461	1.05 (1.03 to 1.06)	10,141	0.51 (0.5 to 0.52)	−3.35 (−5.06 to −1.62)
HIV-MR-TB	133	0.01 (0.01 to 0.01)	2080	0.1 (0.1 to 0.11)	4.67 (1.36 to 8.09)	99	0.01 (0.01 to 0.01)	1,046	0.05 (0.05 to 0.06)	3.34 (−0.01 to 6.79)
HIV-XDR-TB	0	0 (0 to 0)	39	0 (0 to 0)	14.6 (10.15 to 19.24)	0	0 (0 to 0)	24	0 (0 to 0)	13.68 (9.27 to 18.27)
Socio-demographic index										
High	12,337	7.16 (7.04 to 7.28)	3,749	2.21 (2.15 to 2.27)	−3.83 (−4 to −3.66)	646	0.38 (0.36 to 0.41)	36	0.02 (0.02 to 0.02)	−9.19 (−9.35 to −9.02)
High-middle	92,624	30.52 (30.32 to 30.71)	19,275	8.44 (8.32 to 8.55)	−4.41 (−4.56 to −4.26)	7,095	2.31 (2.26 to 2.36)	424	0.18 (0.17 to 0.2)	−8.15 (−8.4 to −7.89)
Middle	392,183	67.33 (67.12 to 67.54)	167,508	29.73 (29.59 to 29.87)	−2.62 (−2.69 to −2.55)	50,167	8.41 (8.34 to 8.49)	6,977	1.27 (1.25 to 1.3)	−5.67 (−6.25 to −5.07)
Low-middle	560,433	123.17 (122.85 to 123.5)	264,320	45.68 (45.51 to 45.86)	−3.15 (−3.39 to −2.91)	112,033	23.25 (23.11 to 23.38)	24,335	4.25 (4.2 to 4.31)	−5.03 (−5.47 to −4.58)
Low	401,278	157.26 (156.77 to 157.75)	343,704	73.83 (73.58 to 74.07)	−2.73 (−3.01 to −2.45)	124,746	44.89 (44.64 to 45.14)	50,047	10.45 (10.36 to 10.54)	−4.73 (−5.12 to −4.34)
GBD regions										
High-income Asia Pacific	1779	5 (4.8 to 5.19)	195	0.82 (0.74 to 0.89)	−5.59 (−5.7 to −5.48)	187	0.58 (0.51 to 0.64)	7	0.03 (0.02 to 0.04)	−9.31 (−9.69 to −8.93)
Central Asia	8,303	33 (32.3 to 33.71)	4,097	14.92 (14.48 to 15.37)	−2.17 (−2.45 to −1.89)	1,494	5.34 (5.1 to 5.59)	349	1.19 (1.09 to 1.29)	−5.27 (−5.82 to −4.71)
East Asia	178,569	52.87 (52.62 to 53.11)	27,840	10.72 (10.6 to 10.85)	−4.96 (−5.14 to −4.78)	21,472	6.18 (6.1 to 6.26)	468	0.19 (0.17 to 0.2)	−10.6 (−10.89 to −10.31)
South Asia	487,262	111.68 (111.36 to 111.99)	196,199	38.58 (38.41 to 38.75)	−3.86 (−4.03 to −3.69)	90,934	19.67 (19.54 to 19.79)	15,890	3.25 (3.2 to 3.3)	−5.61 (−5.71 to −5.51)
Southeast Asia	179,202	104.35 (103.87 to 104.83)	97,701	56.65 (56.3 to 57.01)	−2.04 (−2.29 to −1.79)	31,582	18.1 (17.9 to 18.29)	4,558	2.7 (2.63 to 2.77)	−5.84 (−6.02 to −5.66)
Australasia	161	3.49 (3.14 to 3.84)	90	1.46 (1.27 to 1.65)	−3 (−3.11 to −2.9)	2	0.03 (−0.02 to 0.08)	0	0 (0 to 0)	NA
Caribbean	5,086	44.37 (43.31 to 45.44)	2,519	21.91 (21.22 to 22.6)	−2.43 (−2.52 to −2.34)	1,291	10.66 (10.17 to 11.16)	333	2.87 (2.65 to 3.1)	−4.26 (−4.41 to −4.11)
Central Europe	3,316	11.38 (11.05 to 11.71)	684	3.88 (3.68 to 4.09)	−3.53 (−3.61 to −3.45)	206	0.72 (0.63 to 0.81)	17	0.1 (0.07 to 0.14)	−7.92 (−8.68 to −7.16)
Eastern Europe	12,236	23.85 (23.43 to 24.27)	3,508	9.67 (9.36 to 9.99)	−1.46 (−1.91 to −0.99)	337	0.64 (0.58 to 0.7)	49	0.13 (0.1 to 0.15)	−4.92 (−6.13 to −3.69)
Western Europe	4,042	5.63 (5.46 to 5.79)	1750	2.61 (2.51 to 2.72)	−2.19 (−2.36 to −2.01)	85	0.14 (0.12 to 0.16)	10	0.01 (0.01 to 0.02)	−7.91 (−8.16 to −7.67)
Andean Latin America	12,700	85.03 (83.62 to 86.44)	3,196	17.76 (17.21 to 18.3)	−5.67 (−5.98 to −5.35)	2,551	16.56 (15.98 to 17.13)	172	0.95 (0.85 to 1.04)	−9.19 (−9.38 to −9)
Central Latin America	8,937	13.87 (13.6 to 14.14)	2,734	4.28 (4.14 to 4.42)	−4.4 (−4.68 to −4.12)	1,638	2.48 (2.37 to 2.59)	162	0.26 (0.23 to 0.29)	−7.54 (−8.02 to −7.06)
Southern Latin America	1,640	11.11 (10.67 to 11.56)	751	5.12 (4.84 to 5.39)	−2.91 (−3.1 to −2.71)	163	1.01 (0.9 to 1.13)	23	0.15 (0.11 to 0.18)	−6.14 (−6.22 to −6.07)
Tropical Latin America	8,610	16.14 (15.81 to 16.48)	3,087	6.17 (5.96 to 6.37)	−4.56 (−5.2 to −3.91)	1,417	2.7 (2.57 to 2.82)	163	0.32 (0.29 to 0.36)	−7.16 (−7.36 to −6.96)
North Africa and Middle East	54,491	38.07 (37.75 to 38.39)	22,158	12.15 (11.99 to 12.31)	−3.36 (−3.61 to −3.11)	7,675	4.99 (4.88 to 5.1)	1767	0.98 (0.94 to 1.02)	−4.84 (−5.11 to −4.58)
High-income North America	512	0.82 (0.76 to 0.88)	520	0.79 (0.73 to 0.84)	0.99 (0.51 to 1.47)	44	0.07 (0.06 to 0.09)	5	0 (0 to 0.01)	−9.35 (−9.81 to −8.9)
Oceania	1,432	55.29 (53.14 to 57.43)	2,260	45.31 (43.84 to 46.78)	−0.5 (−0.63 to −0.37)	272	9.64 (8.82 to 10.46)	311	5.8 (5.34 to 6.27)	−1.11 (−1.55 to −0.66)
Central Sub-Saharan Africa	75,447	273.54 (271.6 to 275.49)	88,536	148.83 (147.87 to 149.8)	−1.94 (−2.27 to −1.6)	23,650	76.91 (75.96 to 77.85)	9,819	16.13 (15.83 to 16.42)	−4.67 (−5.34 to −4)
Eastern Sub-Saharan Africa	182,772	189.25 (188.37 to 190.12)	154,491	85.9 (85.48 to 86.33)	−3.02 (−3.45 to −2.58)	67,072	64.22 (63.74 to 64.71)	21,450	11.64 (11.49 to 11.79)	−5.69 (−6.22 to −5.15)
Southern Sub-Saharan Africa	73,007	353.93 (351.37 to 356.49)	46,963	195.77 (194.01 to 197.54)	−1.49 (−2.22 to −0.77)	6,968	33.05 (32.28 to 33.81)	3,985	16.63 (16.13 to 17.14)	−2.93 (−4.63 to −1.19)
Western Sub-Saharan Africa	160,097	167.99 (167.16 to 168.81)	139,767	63.52 (63.19 to 63.85)	−3.02 (−3.37 to −2.68)	35,808	34.9 (34.54 to 35.25)	22,331	9.71 (9.59 to 9.84)	−3.94 (−4.41 to −3.46)

**Figure 1:**
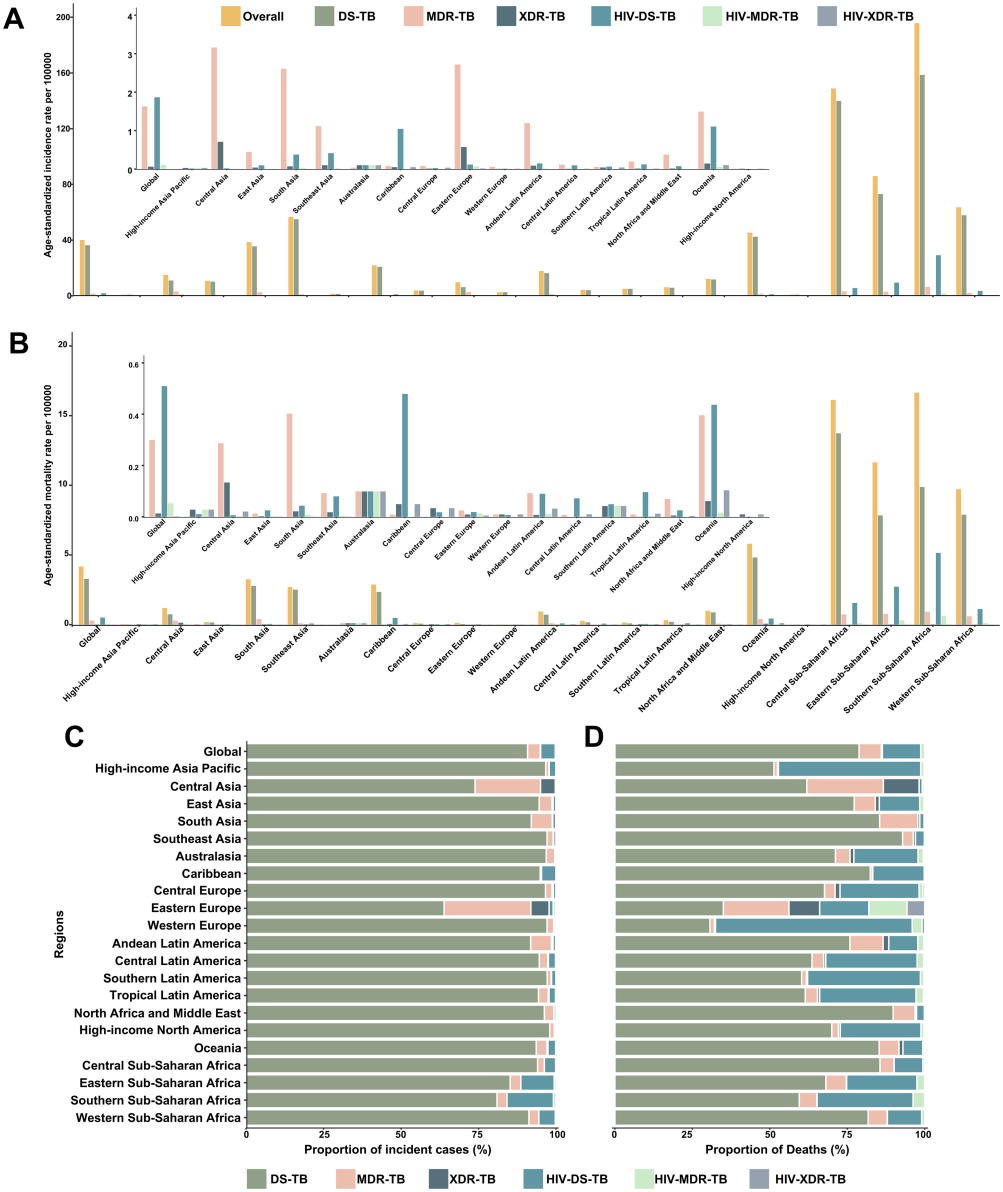
Age-standardized incidence and mortality rates of tuberculosis, and proportions of incident cases and deaths contributed by each type of tuberculosis, globally and for 21 GBD regions, 2021. Age-standardized incidence rates **(A)** and mortality rates **(B)** of each type of tuberculosis. Proportions of incident cases **(C)** and deaths **(D)** accounted for by each type of tuberculosis. DS-TB = Drug-susceptible tuberculosis. MDR-TB = Multidrug-resistant tuberculosis without extensive drug resistance. XDR-TB = Extensively drug-resistant tuberculosis. HIV-DS-TB = HIV-infected drug-susceptible tuberculosis. HIV-MDR-TB = HIV-infected multidrug-resistant tuberculosis without extensive drug resistance. HIV-XDR-TB = HIV-infected extensively drug-resistant tuberculosis.

In 2021, regions of Sub-Saharan Africa had the highest ASIR and ASMR for TB among children and adolescents under 15 years old (Figures 1A,B). From 1990 to 2021, the ASIR declined in all GBD regions, except for high-income North America, which exhibited an upward trend. Likewise, the ASMR decreased across all GBD regions during this period (Table 1). Across all the 21 GBD regions, most TB cases were DS-TB without HIV infection (Figure 1C). Notably, MDR-TB without HIV infection also made a significant contribution in Eastern Europe and Central Asia. While DS-TB without HIV infection accounted for the largest proportion of TB-related deaths across most GBD regions, the deadly impact of DS-TB co-infected with HIV should not be overlooked, especially in Western Europe and high-income Asia Pacific regions (Figure 1D).

### 3.2 National levels

**Figure 2:**
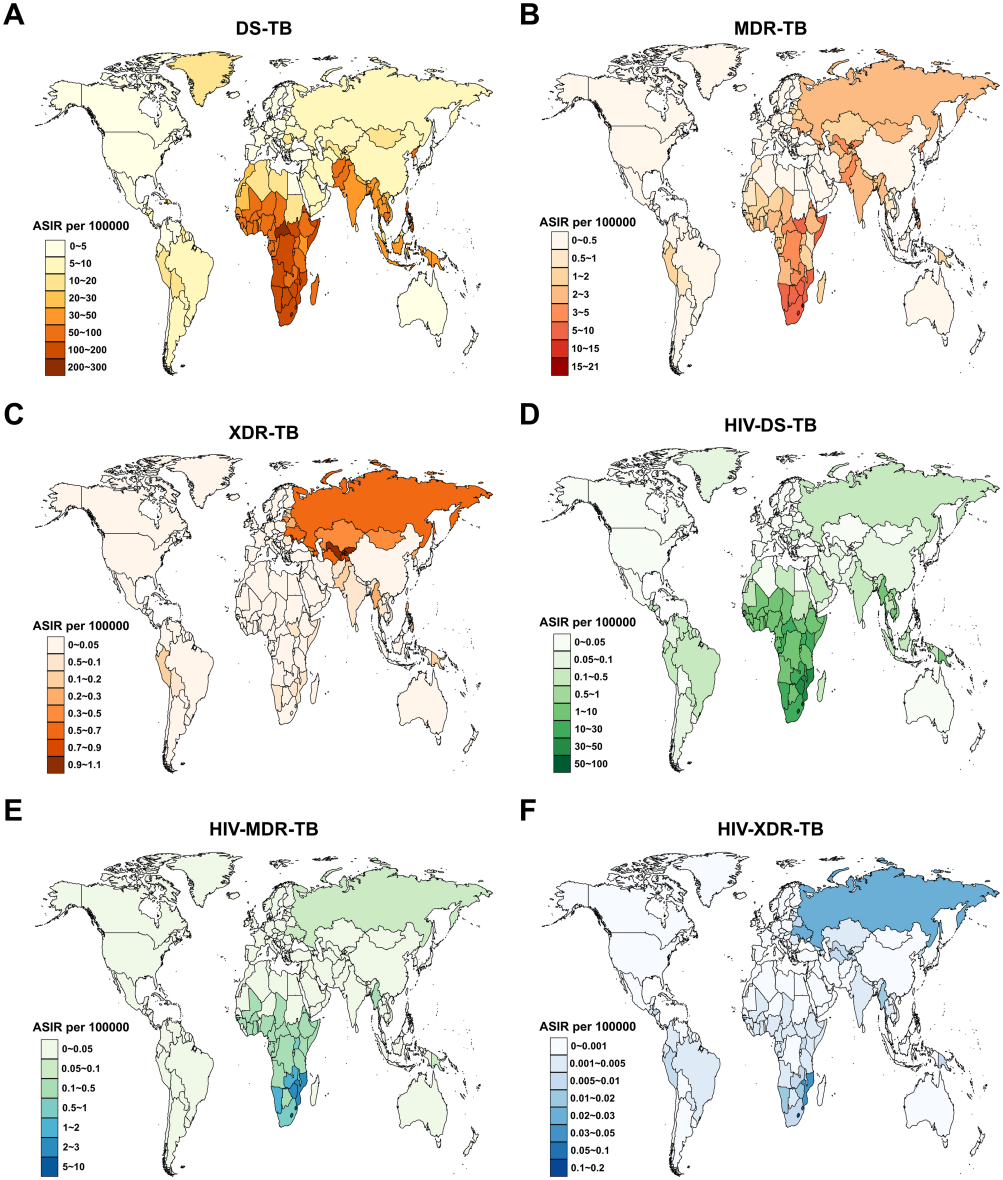
Age-standardized incidence rate (ASIR) for tuberculosis among children and adolescents under 15 years old across 204 countries and territories in 2021. **(A)** DS-TB; **(B)** MDR-TB; **(C)** XDR-TB; **(D)** HIV-DS-TB; **(E)** HIV-MDR-TB; **(F)** HIV-XDR-TB. DS-TB = Drug-susceptible tuberculosis. MDR-TB = Multidrug-resistant tuberculosis without extensive drug resistance. XDR-TB = Extensively drug-resistant tuberculosis. HIV-DS-TB = HIV-infected drug-susceptible tuberculosis. HIV-MDR-TB = HIV-infected multidrug-resistant tuberculosis without extensive drug resistance. HIV-XDR-TB = HIV-infected extensively drug-resistant tuberculosis.

In 2021, the highest ASIR for DS-TB among children and adolescents under 15 years old was in Lesotho (257.6 per 100,000 population; Figure 2A), for MDR-TB in Eswatini (20.0 per 100,000 population; Figure 2B), for XDR-TB in the Republic of Moldova (1.0 per 100,000 population; Figure 2C), for HIV-DS-TB in Lesotho (98.9 per 100,000 population; Figure 2D), for HIV-MDR-TB in Lesotho (9.1 per 100,000 population; Figure 2E), and for HIV-XDR-TB in Lesotho (0.1 per 100,000 population; Figure 2F). On the other hand, the highest ASMR for DS-TB was in the Central African Republic (53.0 per 100,000 population; Supplementary Figure S1A), for MDR-TB in Somalia (3.4 per 100,000 population; Supplementary Figure S1B), for XDR-TB in the Republic of Tajikistan (0.3 per 100,000 population; Supplementary Figure S1C), for HIV-DS-TB in Lesotho (41.8 per 100,000 population; Supplementary Figure S1D), for HIV-MDR-TB in Lesotho (6.0 per 100,000 population; Supplementary Figure S1E), and for HIV-XDR-TB in Lesotho (0.08 per 100,000 population; Supplementary Figure S1F).

**Figure 3:**
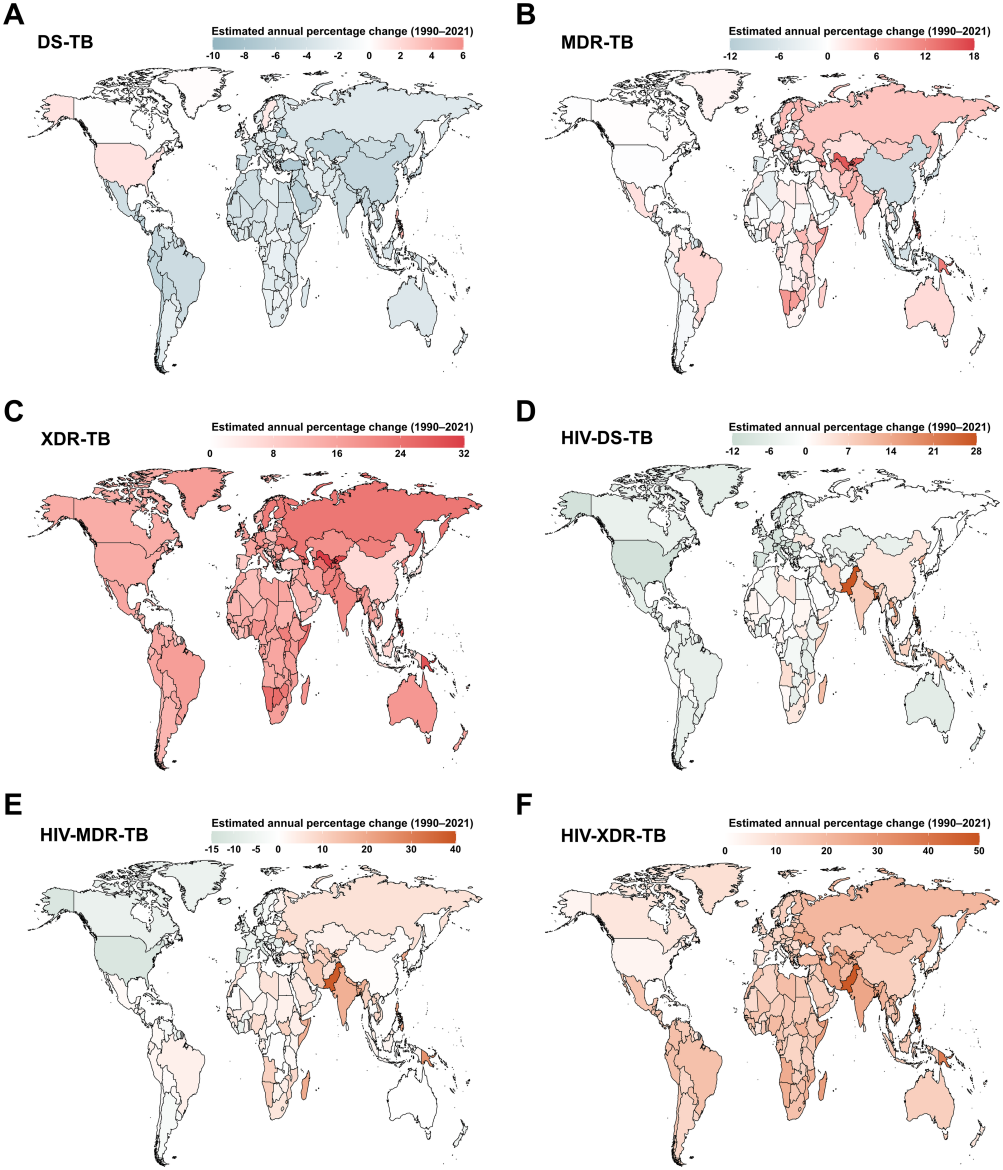
Estimated annual percentage changes in age-standardized incidence rate (ASIR) for tuberculosis among children and adolescents under 15 years old across 204 countries and territories from 1990 to 2021. **(A)** DS-TB; **(B)** MDR-TB; **(C)** XDR-TB; **(D)** HIV-DS-TB; **(E)** HIV-MDR-TB; **(F)** HIV-XDR-TB. DS-TB = Drug-susceptible tuberculosis. MDR-TB = Multidrug-resistant tuberculosis without extensive drug resistance. XDR-TB = Extensively drug-resistant tuberculosis. HIV-DS-TB = HIV-infected drug-susceptible tuberculosis. HIV-MDR-TB = HIV-infected multidrug-resistant tuberculosis without extensive drug resistance. HIV-XDR-TB = HIV-infected extensively drug-resistant tuberculosis.

Between 1990 and 2021, an increase in the ASIR of DS-TB was observed in only 6 out of 204 countries and territories, namely the Philippines, United States of America, Sweden, United Kingdom, Greenland, and Bulgaria (Figure 3A). Globally, nearly half of all countries and territories experienced an increase in the ASIR of MDR-TB, with the largest rise in Uzbekistan (average annual change 16.01% [95% CI 12.37 to 19.76]; Figure 3B). The ASIR of XDR-TB also tended to increase across all 204 countries and territories (Figure 3C). Pakistan reported the most rapid increases in the ASIR of HIV-DS-TB (average annual change 27.4% [95% CI 24.57 to 30.29]; Figure 3D), HIV-MDR-TB (38.23% [95% CI 32.86 to 43.8]; Figure 3E), and HIV-XDR-TB (48.37% [95% CI 40.74 to 56.41]; Figure 3F).

Regarding estimated annual percentage changes in the ASMR of DS-TB at the country level, only Zimbabwe showed a rising trend during this period (Supplementary Figure S2A). The largest increases in ASMR of both MDR-TB and XDR-TB were observed in Papua New Guinea, with average annual change 11.35% (95% CI 9.56 to 13.17) and 33.74% (95% CI 24.64 to 43.5), respectively (Supplementary Figures S2B,C). Additionally, Pakistan reported the most rapid increases in the ASMR of HIV-DS-TB (average annual change 30.79% [95% CI 27.06 to 34.62]; Supplementary Figure S2D), HIV-MDR-TB (46.06% [95% CI 38.95 to 53.54]; Supplementary Figure S2E), and HIV-XDR-TB (41.54% [95% CI 33.2 to 50.4]; Supplementary Figure S2F).

### 3.3 The association between ASR and SDI

The ASIR and ASMR of TB were higher in regions with lower SDI compared to those with higher SDI, but this gap has been narrowing over time (Supplementary Figures S3A,B). Between 1990 and 2021, both the ASIR and ASMR of TB significantly decreased across all five SDI regions. Similarly, at the regional level, the ASIR and ASMR for TB declined exponentially as SDI increased (Supplementary Figures S3C,D). However, despite this overall trend, Southern and Central sub-Saharan Africa had higher-than-expected ASIR and ASMR of TB based on their SDI during this period (Supplementary Figures S3C,D).

### 3.4 Age pattern

As expected, the proportion of drug-resistant TB or TB co-infected with HIV contributing to overall TB incidence and deaths increased with advancing age (Supplementary Figure S4). Notably, half of the new TB cases globally occur in children under 5 years of age, and this proportion increases as SDI levels decrease (Supplementary Figure S5A). Additionally, children under 5 account for more than 75% of TB-related deaths worldwide, with this figure rising to 80% in low-SDI regions (Supplementary Figure S5B). Furthermore, the TB burden in this youngest age group decreases between 1990 and 2021 in lower-SDI regions, whereas it remains unchanged in regions with higher SDI (Supplementary Figure S5).

### 3.5 Prediction through 2040

**Figure 4:**
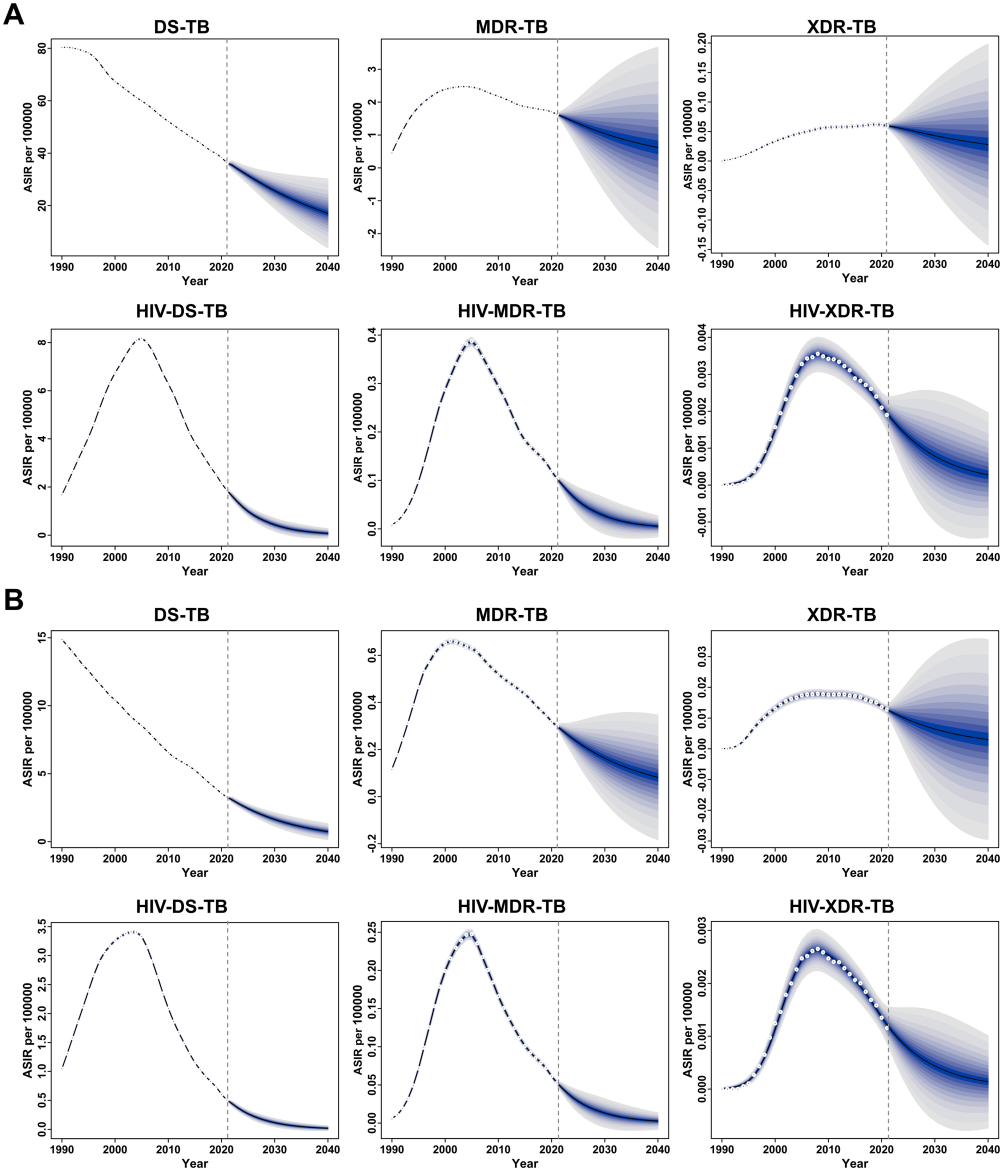
Global temporal trends in the age-standardized incidence rate (ASIR) **(A)** and age-standardized mortality rate (ASMR) **(B)** for specific forms of tuberculosis among children and adolescents under 15 years old from 1990 to 2021, with projections through 2040. The blue shaded area represents the 95% uncertainty interval (UI), highlighting the upper and lower bounds.

Fortunately, both the ASIR and ASMR for all six forms of TB among children and adolescents under 15 years old are projected to decline between 2021 and 2040 (Figure 4). The ASIR for DS-TB, MDR-TB, XDR-TB, HIV-DS-TB, HIV-MDR-TB, and HIV-XDR-TB is expected to decrease from 36.35, 1.63, 0.06, 1.86, 0.10, and 0.002 in 2021 to 17.04, 0.62, 0.03, 0.08, 0.01, and 0.0002 in 2040, respectively (Figure 4A). Similarly, the ASMR for DS-TB, MDR-TB, XDR-TB, HIV-DS-TB, HIV-MDR-TB, and HIV-XDR-TB is projected to decline from 3.28, 0.30, 0.01, 0.51, 0.05, and 0.001 in 2021 to 0.74, 0.08, 0.003, 0.02, 0.003, and 0.0001 in 2040, respectively (Figure 4B).

## 4 Discussion

In 2021, we documented 799,047 new TB cases among children and adolescents under 15 years old globally, underscoring the persistent and alarming burden of TB among this susceptible age group. Despite a global decline in ASIR and ASMR for overall TB from 1990 to 2021, significant disparities remain, particularly in regions with low SDI, highlighting an urgent need for focused public health interventions. Additionally, the trends in drug-resistant TB, especially XDR cases, warrant particular attention. The rise in drug-resistant TB cases from 1990 to 2021 is alarming, as it complicates treatment protocols and further endangers this already susceptible population (17). Our data indicate that the majority of pediatric TB cases are attributed to DS-TB without HIV infection; however, the significant contributions from MDR-TB and the lethal impact of co-infections with HIV cannot be overlooked (18, 19). Our analysis highlights significant age-related trends in TB incidence and mortality, underscoring that children under 5 years bear a disproportionate burden, accounting for more than half of new cases and 75% of TB-related deaths. This disparity is particularly pronounced in low-SDI regions, where TB rates are on the rise. Young children under five are at higher risk of undiagnosed TB and developing severe disease compared to older children, emphasizing the critical need for age-specific approaches in TB prevention, diagnosis, and treatment, particularly for this susceptible population (20).

Regions of Sub-Saharan Africa remains the epicenter of pediatric TB, with the highest ASIR and ASMR observed in Lesotho and the Central African Republic. WHO data indicate that approximately 17 of the 30 countries with the highest prevalence of pediatric TB are in the African region, accounting for nearly one-third of global TB cases in children under 15 (21). Several factors exacerbate the spread and impact of TB in this region. First, Sub-Saharan Africa has the highest global rates of HIV, a major risk factor for TB, particularly in children. HIV compromises the immune system, making children more susceptible to TB (22). Second, widespread poverty and food insecurity lead to malnutrition, further weakening immune defenses and increasing children’s vulnerability to the disease (23). Third, high population densities and overcrowded living conditions facilitate TB transmission, especially in environments where children are frequently in contact with infected adults (24). Fourth, limited access to healthcare services reduces Bacille Calmette-Guérin (BCG) vaccination coverage and delays the detection and treatment of TB in children (25). Lastly, delayed or incomplete TB treatment, often resulting from inadequate healthcare infrastructure or drug supply interruptions, contributes to the rise of drug-resistant TB strains, which are much harder to treat (26). These challenges underscore the urgent need for increased resource allocation and tailored public health strategies to address the specific epidemiological challenges in this region.

The association between TB burden and SDI reinforces the notion that socio-economic factors play a crucial role in TB epidemiology. While we observed an overall decrease in both ASIR and ASMR across all SDI regions, the gap between lower and higher SDI areas persists, particularly in Southern and Central sub-Saharan Africa, where TB rates remain disproportionately high. This suggests that economic improvements alone may not be sufficient; tailored interventions that consider local contexts are critical. In regions with high HIV prevalence, integrating TB and HIV services in healthcare centers is vital to ensure simultaneous testing and treatment, reducing the risk of TB co-infection in children with HIV (27). For children with HIV, prompt initiation of antiretroviral therapy (ART) further lowers the risk of developing TB, making the expansion of ART coverage and ensuring adherence key to reducing the pediatric TB burden (28). Additionally, strengthening surveillance systems to monitor and manage MDR- and XDR-TB, especially among children, is crucial for early detection and effective treatment (29). Furthermore, expanding TB prevention programs, such as increasing BCG vaccination coverage and providing isoniazid preventive therapy (IPT) for exposed children—particularly those with HIV or under 5 years old—remains essential (30, 31). Training healthcare workers to recognize pediatric TB, which often presents with non-specific symptoms, is equally important. Innovative, cost-effective, and child-friendly diagnostic tools, such as CRISPR-based biosensing, show promise for rapid and sensitive detection of pediatric TB (32, 33).

Over the past decades, national tuberculosis programs in high-burden regions have increasingly focused on addressing the challenges of childhood tuberculosis (34). In 2012, World TB Day highlighted children for the first time, marking a pivotal shift in global attention. This focus is expected to grow as the WHO Global Tuberculosis Programmer’s ambitious post-2015 TB control strategy aims to involve the entire healthcare system, including maternal and child health services, to better combat TB in young populations (35). Encouragingly, our projections through 2040 suggest a potential decline in both ASIR and ASMR across all forms of TB among children and adolescents under 15 years of age. This positive outlook emphasizes the impact of ongoing public health initiatives, including vaccination, improved diagnostics, and treatment protocols. However, to realize these projections, sustained investment in TB control programs is essential, particularly in high-burden areas. One of the most effective strategies for lowering TB rates is widespread vaccination. The BCG vaccine, administered at birth, remains the foundation of TB prevention in children, especially in high-incidence countries (36). Several new vaccines, such as the M72/AS01E, are currently undergoing clinical trials and have demonstrated efficacy in adults. If proven effective in children, these vaccines could significantly reduce TB incidence (37). Our projections should be viewed as a baseline forecast that assumes continuation of present trends in TB control. By design, the BAPC model does not include any hypothetical future interventions or disruptions. Real-world uncertainties, such as reduced funding due to economic downturns, emergent health crises (e.g., new pandemics), or political instability in high-burden regions, could impede progress. Conversely, accelerated declines could occur if innovations like the M72/AS01E vaccine or CRISPR-based diagnostics are scaled up in pediatric populations. However, our model’s inability to incorporate such unimplemented interventions necessitates cautious interpretation of projections.

Training community health workers to screen children for TB symptoms and refer them for testing is another impactful measure, especially in remote areas with limited healthcare access (38). While TB treatment is generally effective, managing pediatric TB presents challenges due to long treatment durations, adherence difficulties, and the increasing threat of drug-resistant TB. The WHO has recommended shorter regimens, with treatments as brief as 4 months for certain forms of TB, which should be scaled up globally (39). Additionally, child-friendly drug formulations, such as flavored dispersible tablets, can improve adherence to treatment (40). Routine drug susceptibility testing (DST) in pediatric TB cases, especially in high-burden settings, is crucial for identifying drug-resistant strains early. Expanding access to newer drugs like bedaquiline and delamanid, which are effective against drug-resistant TB and have been approved for pediatric use, could significantly reduce mortality from these resistant strains (41).

The current study has several limitations. First, the accuracy of our estimates may be affected by underreporting, particularly in low-SDI regions where weak healthcare infrastructure and inadequate TB diagnosis and reporting systems are prevalent. This underreporting is likely to lead to an underestimation of TB incidence and mortality, especially among pediatric populations, where cases often remain undiagnosed. Second, while our study highlights the growing burden of drug-resistant TB, including MDR- and XDR-TB, data on pediatric drug-resistant TB are limited. Many countries do not routinely conduct drug resistance testing in children, resulting in potential underreporting and limiting the generalizability of our findings on this population. Third, though this study emphasizes the significant TB burden in low-SDI regions, it does not fully capture the regional disparities in healthcare access, which heavily influence TB prevention, diagnosis, and treatment outcomes. The inequitable access to healthcare could lead to uneven progress in TB reduction across different areas. Finally, while our projections indicate a decline in TB incidence and mortality through 2040, these estimates are based on current trends and interventions. Any shifts in global TB control strategies, healthcare infrastructure, or developments in drug-resistant TB could alter these projections, potentially leading to discrepancies between expected and actual outcomes.

## 5 Conclusion

In conclusion, this study highlights the pressing need for targeted public health interventions tailored to the unique epidemiological patterns of TB in children and adolescents. Addressing the complex interplay of socio-economic factors, drug resistance, and age-specific vulnerabilities is crucial to achieving significant reductions in TB incidence and mortality in this susceptible population. Continued surveillance and research are vital to inform policy and practice, ensuring that the burden of TB among children and adolescents is effectively mitigated in the coming decades.

## Data Availability

The original contributions presented in the study are included in the article/Supplementary material, further inquiries can be directed to the corresponding author/s.

## Footnotes

^1^ http://ghdx.healthdata.org/gbd-results-tool

^2^ https://seer.cancer.gov/stdpopulations/world.who.html
