# Supplementary material for: Global burden and trend of tuberculosis in children and adolescents (under 15 years old) from 1990 to 2021, with projections to 2040
Source: Front Public Health. 2025 Jun 25;13:1578658. doi: 10.3389/fpubh.2025.1578658 (PMC12237893; doi:10.3389/fpubh.2025.1578658)
Supplement: Supplementary file 1 [file Data_Sheet_1.docx]

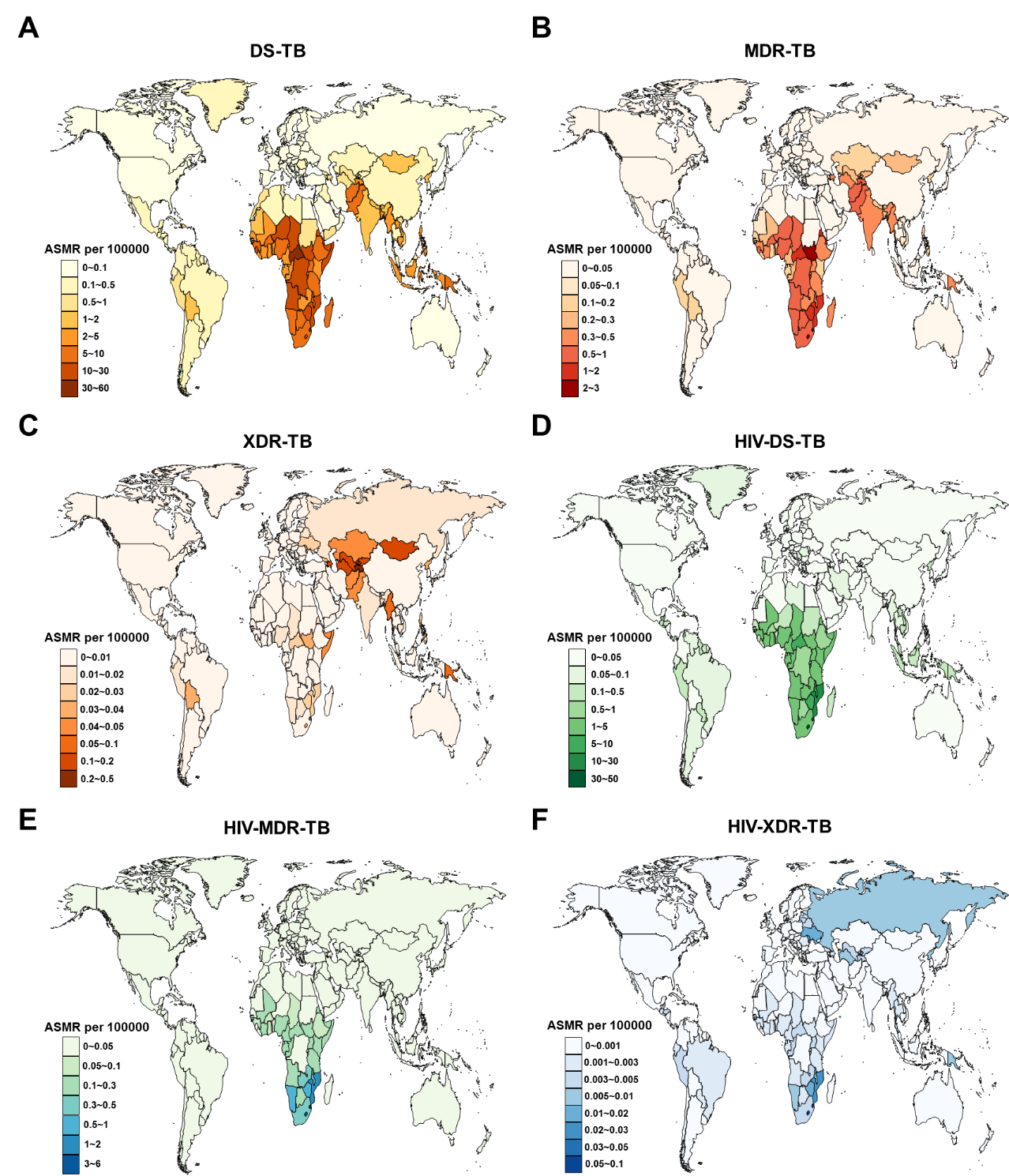


**Supplementary Figure S1. Age-standardized mortality rate (ASMR) for tuberculosis among children and adolescents under 15 years old across 204 countries and territories in 2021.**

(A) DS-TB; (B) MDR-TB; (C) XDR-TB; (D) HIV-DS-TB; (E) HIV-MDR-TB; (F) HIV-XDR-TB. DS-TB=Drug-susceptible tuberculosis. MDR-TB=Multidrug-resistant tuberculosis without extensive drug resistance. XDR-TB=Extensively drug-resistant tuberculosis. HIV-DS-TB=HIV-infected drug-susceptible tuberculosis. HIV-MDR-TB=HIV-infected multidrug-resistant tuberculosis without extensive drug resistance. HIV-XDR-TB=HIV-infected extensively drug-resistant tuberculosis.


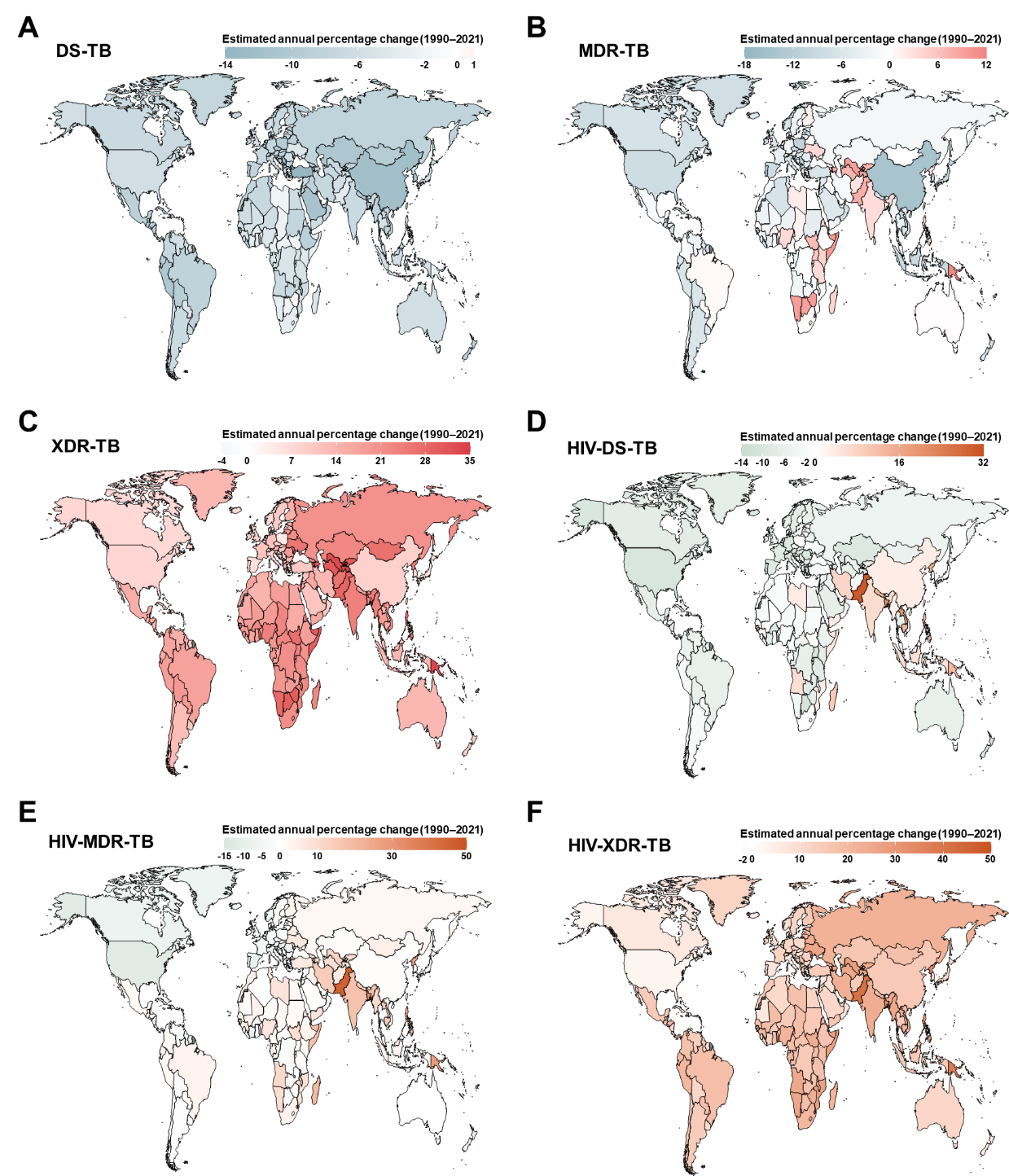


**Supplementary Figure S2. Estimated annual percentage changes in age-standardized mortality rate (ASMR) for tuberculosis among children and adolescents under 15 years old across 204 countries and territories from 1990 to 2021.**

(A) DS-TB; (B) MDR-TB; (C) XDR-TB; (D) HIV-DS-TB; (E) HIV-MDR-TB; (F) HIV-XDR-TB. DS-TB=Drug-susceptible tuberculosis. MDR-TB=Multidrug-resistant tuberculosis without extensive drug resistance. XDR-TB=Extensively drug-resistant tuberculosis. HIV-DS-TB=HIV-infected drug-susceptible tuberculosis. HIV-MDR-TB=HIV-infected multidrug-resistant tuberculosis without extensive drug resistance. HIV-XDR-TB=HIV-infected extensively drug-resistant tuberculosis.


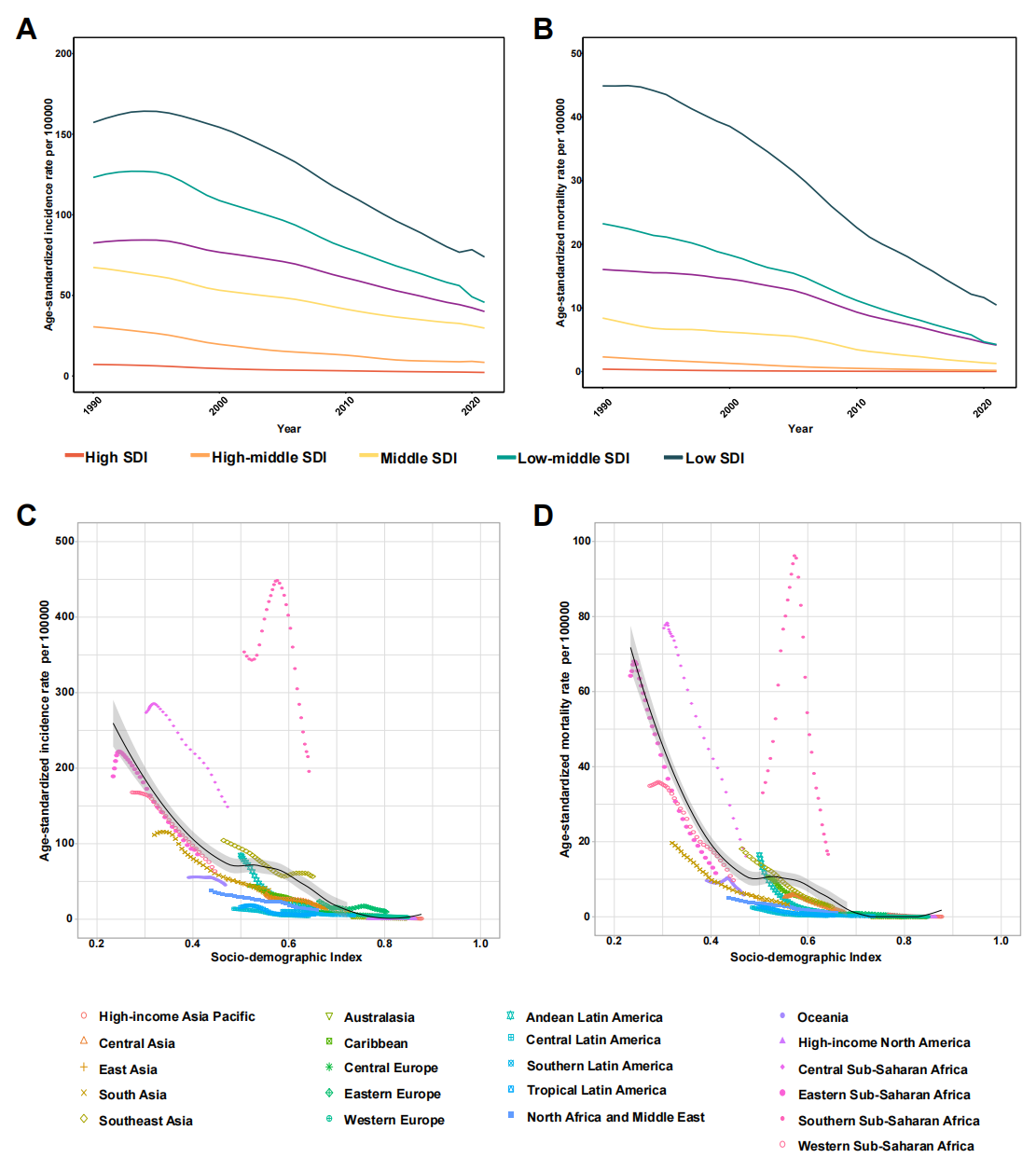


**Supplementary Figure S3.** **Association between age-standardized incidence and mortality rates of tuberculosis with the Sociodemographic Index.**

Trends in age-standardized incidence rates (ASIR) (A) and age-standardized mortality rates (ASMR) (B) of tuberculosis among children and adolescents under 15 years from 1990 to 2021, categorized by Sociodemographic Index (SDI). Trends in ASIR (C) and ASMR (D) across the 21 Global Burden of Disease (GBD) regions, stratified by SDI, during this period.


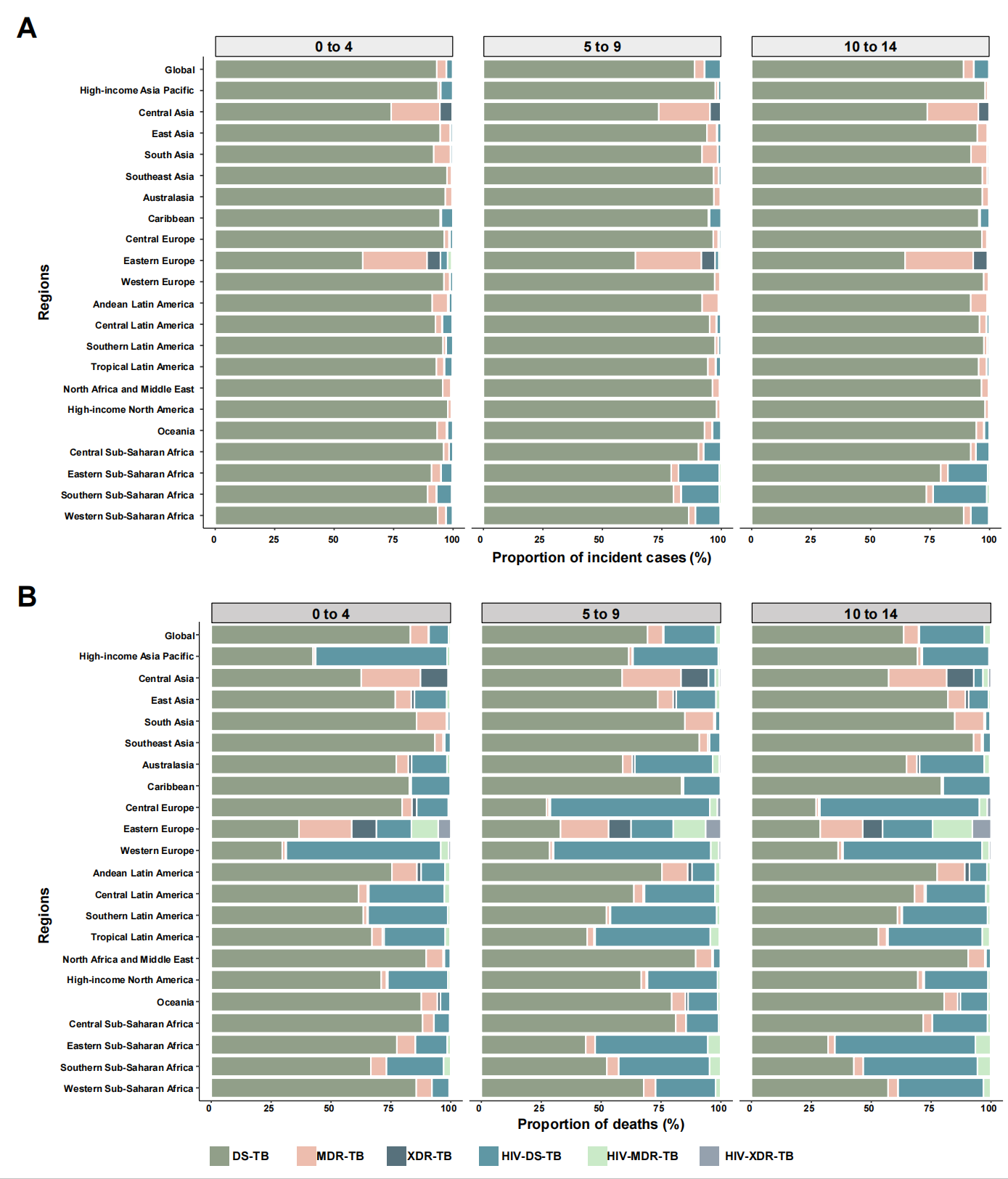


**Supplementary Figure S4. Proportions of incident cases (A) and deaths (B) contributed by each type of tuberculosis by age group, globally and for 21 GBD regions, 2021.**

DS-TB=Drug-susceptible tuberculosis. MDR-TB=Multidrug-resistant tuberculosis without extensive drug resistance. XDR-TB=Extensively drug-resistant tuberculosis. HIV-DS-TB=HIV-infected drug-susceptible tuberculosis. HIV-MDR-TB=HIV-infected multidrug-resistant tuberculosis without extensive drug resistance. HIV-XDR-TB=HIV-infected extensively drug-resistant tuberculosis.

**
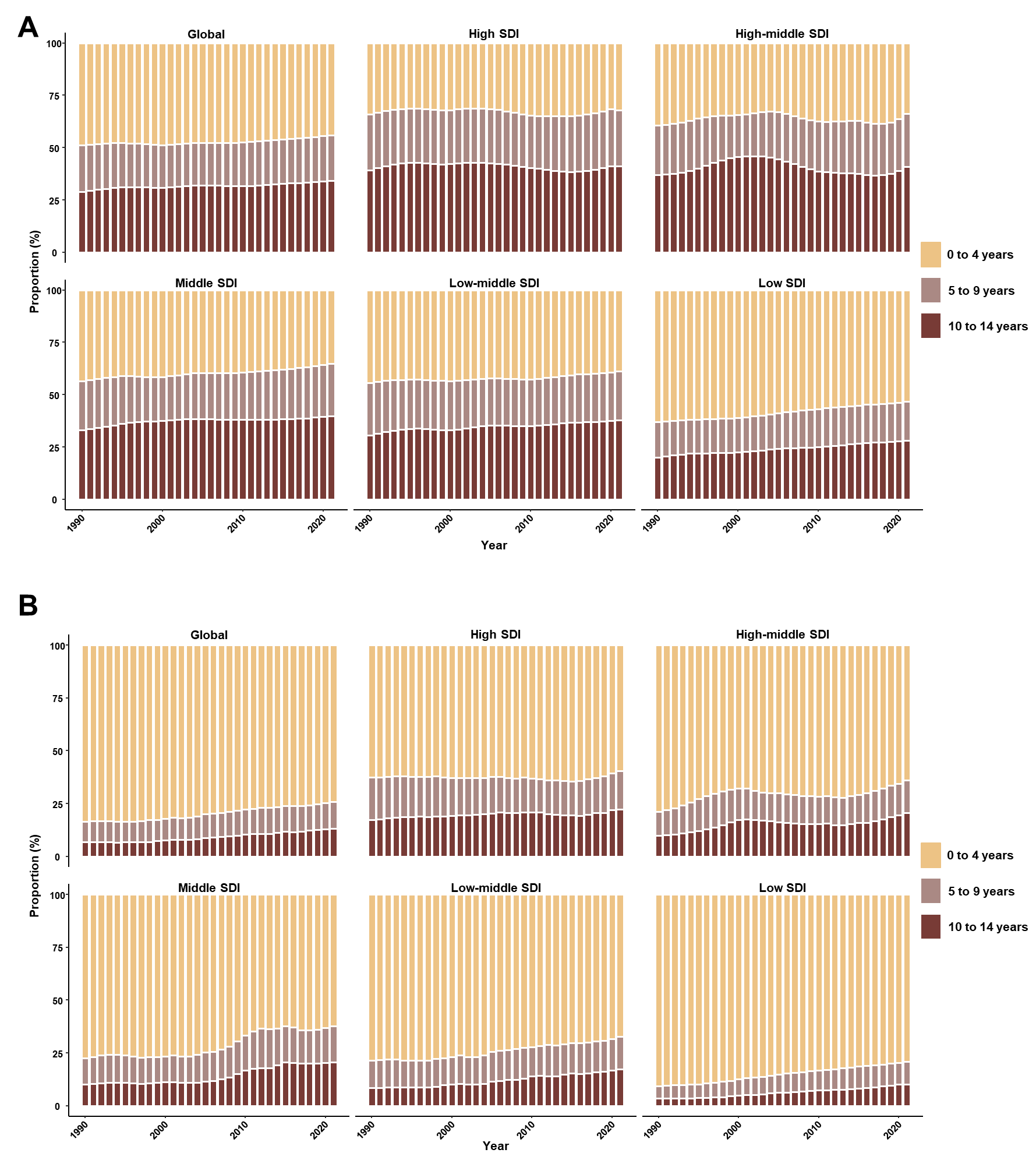
**

**Supplementary Figure S5. Proportion of tuberculosis incidence (A) and deaths (B) among children and adolescents under 15 years old, attributable to specific age groups, both globally and within regions with different Sociodemographic Index (SDI) levels, from 1990 to 2021.**
